# Comparison of facial muscle activity between younger and older Japanese adults: A cross-sectional study

**DOI:** 10.1371/journal.pone.0357143

**Published:** 2026-10-21

**Authors:** Rumiko Okamoto, Kazutaka Adachi, Katsuyoshi Mizukami

**Affiliations:** 1 Graduate School of Comprehensive Human Sciences, University of Tsukuba, Tsukuba, Ibaraki, Japan; 2 SBC Tokyo Medical University, Urayasu, Japan; Lyon College, UNITED STATES OF AMERICA

## Abstract

A relationship has been reported between facial expressions and mental health. Surface electromyography (sEMG) is a standard modality used in clinical and psychosocial research to quantitatively assess facial muscle activity. However, limited information is currently available on age-associated differences in facial muscle activity. Therefore, we investigated facial muscle function by measuring activity during established voluntary facial movement tasks and comparing facial muscle activity between younger and older Japanese adults. This was a cross-sectional study that recruited participants using snowball sampling. The present results showed that during the neutral facial condition, older adults (N = 23; F: 19/ M: 4) exhibited significantly higher muscle activity than younger adults. In contrast, during other facial tasks, younger adults (N = 20; F: 9/ M: 11) showed significantly greater facial muscle activity. The increased muscle activity observed in the older group when expressing neutral facial expressions may be due to age-associated changes in muscle tone mediated by the sympathetic nervous system, similar to those observed in skeletal muscles. The decreased muscle activity during voluntary facial movements in the older group may also be related to aging.

## 1. Introduction

Recent studies have reported that declines in oral functions, such as tongue strength and masticatory performance, are associated with frailty and pre-frailty in older adults [1,2]. Tanaka et al. proposed the concept of “oral frailty” to describe multidimensional declines in oral health that may precede physical frailty and functional disability [2]. In addition to nutritional and physical consequences, oral frailty has been associated with reduced social participation, impaired communication, and depressive symptoms in older adults [3,4]. These findings suggest that age-related decline in oral function may extend beyond eating and swallowing and may influence broader communication-related functions.

Communication relies not only on oral function but also on facial motor function. Facial expressions play essential roles in emotional expression, nonverbal communication, and social interaction, which are closely associated with psychological well-being and quality of life in older adults. Previous studies have reported that reduced facial expressivity may negatively affect interpersonal communication and social functioning in both clinical and non-clinical populations [5–8]. In addition, aging has been associated with changes in facial mimicry, emotional expression, and facial movement patterns; however, most previous studies have relied on observational assessments, image analysis, or subjective ratings rather than direct physiological measurements.

Facial muscles, innervated by the facial nerve, contribute to feeding, speech production, and emotional expression [9]. Because facial movements are typically generated through coordinated activation of multiple small and anatomically adjacent muscles, objective assessment of underlying muscle activity remains challenging. Surface electromyography (sEMG) enables detection of subtle facial muscle activation that may not be visible during visual observation alone and has been widely used in both clinical and experimental settings [10–12].

Previous studies have demonstrated that low-level facial muscle activity may be present even during resting conditions [13]. Cotofana et al. compared facial muscle activity between younger and older adults using sEMG and reported age-group differences in muscle activation during instructed facial expressions [14]. However, their assessment focused on muscle activation associated with instructed facial expressions and did not evaluate facial muscle activation during maximal-effort voluntary functional facial movements. Thus, although sEMG has been used to compare facial muscle activity between younger and older adults, facial muscle activation during maximal-effort voluntary facial movements remains insufficiently understood. In particular, objective comparisons of maximal-effort voluntary facial muscle activation during functionally relevant facial movements across age groups are limited. Importantly, evaluating facial muscle function itself — rather than facial expression alone — may provide a more direct and objective measure of differences in neuromuscular function across age groups, with potential implications for the early detection of functional decline and the promotion of healthy aging.

Therefore, the present study aimed to compare maximal-effort voluntary facial muscle activation during facial movements that reflect the primary functional roles of each facial muscle group between younger and older adults using sEMG.

## 2. Materials and methods

### 2.1. Study design and subjects

This cross-sectional study recruited participants through community-based and university-related networks using snowball sampling between August 1 and October 31, 2023. Measurements were conducted at the Tsukuba and Tokyo campuses of the University of Tsukuba between August 28 and December 10, 2023. The final sample size for each group is reported in the Results section.

### 2.2. Inclusion and exclusion criteria

We included participants who lived independently and did not require nursing care. We excluded individuals with diagnosed dementia, severe cognitive impairment that could interfere with understanding task instructions, those requiring nursing care or assistance with activities of daily living, and those with conditions that could affect facial muscle activity or sEMG recordings. These included a history of facial nerve disorders, prior facial surgery, botulinum toxin treatment, facial aesthetic procedures, marked facial asymmetry, or visible scar tissue in areas relevant to electrode placement. No participants met these exclusion criteria during recruitment.

### 2.3 EMG measurements

Facial sEMG signals were recorded to evaluate muscle activity during rest and facial tasks. The following muscles were assessed: frontalis (FRO), corrugator supercilii (CS), orbicularis oculi (OOc), levator labii superioris alaeque nasi (LLSAN), levator labii superioris (LLS), zygomaticus major (ZM), levator anguli oris (LAO), orbicularis oris (OOr), and depressor anguli oris (DAO).

Electrode placement followed the original guidelines proposed by Fridlund and Cacioppo (1986) [15] (Fig 1).

**Fig 1:**
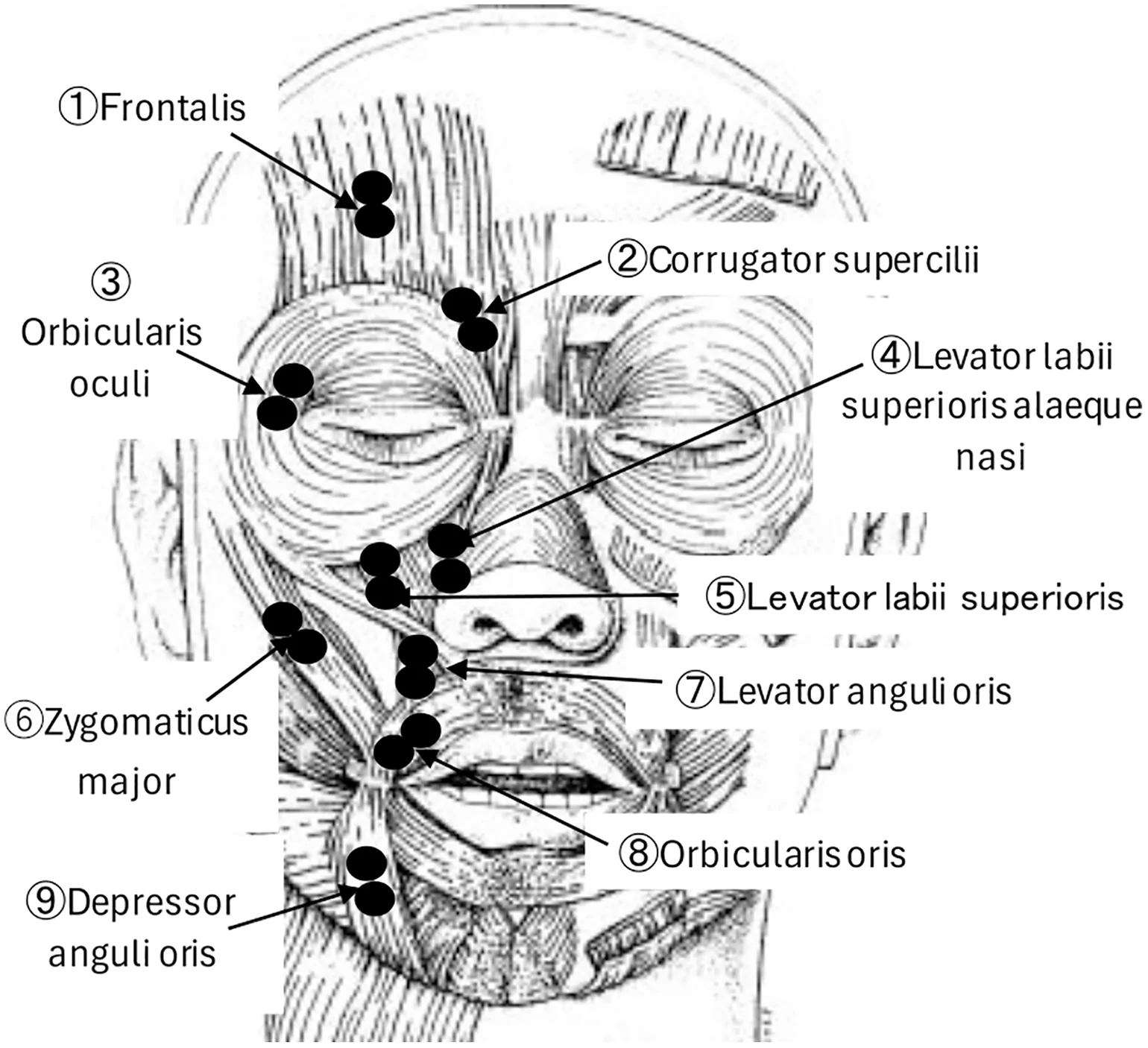
Electrode locations for facial muscles. Based on and modified by Fleilinger et al., histochemical mapping and fiber size analysis of mimic muscles were performed. 1990.

Before electrode placement, the skin was cleaned with alcohol wipes to reduce surface impedance. Paired Ag/AgCl surface electrodes (5 mm diameter) were placed parallel to the muscle fibers with an inter-electrode distance of approximately 10 mm (center-to-center), based on facial sEMG guidelines and adjusted as anatomically necessary to minimize crosstalk between adjacent facial muscles.

Facial sEMG signals were recorded using a multichannel telemetry system (WEB-5000; Nihon Kohden, Tokyo, Japan), which has also been used in our previous facial sEMG study [16]. Signals were recorded using a bipolar configuration, A/D converted via PowerLab/16SP, and analyzed using LabChart software (ADInstruments, Australia). The sampling rate was set at 1,000 Hz, which exceeds the frequency range typically observed in surface EMG signals (approximately 20–450 Hz) and satisfies the Nyquist criterion [15]. The time constant was set at 0.03 seconds, corresponding to a high-pass filter cutoff frequency of approximately 5 Hz. This setting was selected to preserve low-frequency components of facial muscle activity, particularly during resting conditions and low-amplitude contractions, because facial muscles are anatomically small and often produce lower-amplitude EMG signals than larger skeletal muscles.

Participants were instructed to maintain each facial movement for 5 seconds. Root mean square (RMS) amplitudes were calculated from the raw EMG signals using 1-second analysis windows. The selection of a 1-second epoch was consistent with our previously published facial sEMG protocol [16], in which a 1-second segment was selected from a 5-second recording for RMS analysis. Following this previously established protocol, one continuous 1-second epoch showing relatively stable EMG activity during the intended facial movement was selected from each 5-second recording for the primary analysis. sEMG recordings were visually inspected for signal instability and apparent motion artifacts, and simultaneous video recordings were used to confirm successful task performance and maintenance of the intended facial movement during the selected epoch. The same approach was applied across participants. Because of participant burden and the large number of facial movement tasks, a single recording trial was obtained for each task.

Because facial expressions typically involve coordinated activation of multiple muscles, the tasks were designed to assess facial movements that reflect the primary functional roles of each muscle group rather than isolate the activity of a single muscle (Table 1).

**Table 1:** Tasks for facial movement.

No.	Primary Muscle Group	Task	Instruction
1	-	Neutral	Please look at the video camera and keep your face relaxed with a neutral expression.
2	Frontalis muscle	Frontalis muscle contraction	Raise your eyebrows and create horizontal wrinkles on your forehead.
3	Corrugator supercilii muscle	Corrugator supercilii muscle contraction	Lower your eyebrows as much as possible and create wrinkles between your eyebrows.
4	Orbicularis oculi muscle	Orbicularis oculi muscle contraction	Close your eyes as tightly as possible.
5	Levator labii superioris alaeque nasi muscle	Levator labii superioris alaeque nasi muscle contraction	Lift your nostrils and wrinkle your nose as much as possible.
6	levator labii superioris	levator labii superioris muscle contraction	Raise your cheeks straight upward as much as possible.
7	Zygomaticus major muscle	Zygomaticus major muscle contraction	Lift the corners of your mouth to create a broad smile.
8	levator anguli oris	levator anguli oris contraction	Lift your upper lip upward and show your upper gums as much as possible.
9	Orbicularis oris muscle	Orbicularis oris muscle contraction	Close your lips tightly as much as possible.
10	Depressor anguli oris muscle	Depressor anguli oris muscle contraction	Pull the corners of your mouth downward and maintain the movement.

To minimize variability in task performance, all participants received standardized verbal instructions and demonstrations from the same examiner prior to testing. Participants completed practice trials until they understood each facial movement task. Prior to the formal recording trial, participants performed practice contractions to confirm that the intended facial movement was correctly executed and that an adequate level of muscle activation was achieved. The formal recording trial was initiated only after the examiner confirmed that the participant was able to perform the task correctly and consistently. This procedure was intended to minimize within-participant variability in task execution and to ensure that the single recording trial reflected a representative maximal-effort contraction. During data collection, facial movements were simultaneously video-recorded using a digital video camera (HDR-CX670, Sony, Japan) to confirm whether the intended movement was successfully performed. Participants were instructed to perform each facial movement with maximal effort; however, no standardized objective method for verifying maximal effort during facial movements currently exists, which should be addressed in future studies.

Task 1 represented the resting condition and was used as the baseline value for each participant. For Tasks 2–10, RMS values were normalized by dividing each task value by the corresponding resting value. This normalization approach was used to reduce inter-individual variability in absolute facial sEMG amplitudes caused by factors such as subcutaneous tissue characteristics, skin impedance, and minor differences in electrode placement. Because facial muscles are anatomically small and show substantial inter-individual variability, relative changes from resting activity were considered more appropriate for between-group comparisons. However, because significant between-group differences were observed in resting muscle activity for several muscles, this normalization approach may have influenced the magnitude of group differences observed during active tasks. To improve transparency, raw RMS values for active tasks are also provided in S1-S9 Tables in S1 File. These relative changes in muscle activity were subsequently used for statistical analyses.

As a sensitivity analysis, RMS amplitudes were additionally calculated from the entire 5-second recording for each task and muscle, without selecting a specific 1-second epoch. These 5-second RMS values were analyzed using the same statistical procedures as the primary analysis to evaluate whether the observed age-group differences were robust to the method used to define the analysis epoch.

### 2.4. Statistical analysis

Sample size was estimated a priori using G*Power version 3.1.9.2 based on a large effect size (Cohen’s d = 0.80), an alpha level of 0.05, and a statistical power of 0.80. The effect size was determined based on the limited previous literature examining differences in facial muscle activity between younger and older adults [14] and Cohen’s conventional criteria for large effects [17]. Categorical variables were compared using Fisher’s exact test, and normality of continuous data was assessed using the Shapiro–Wilk test.

Depending on data normality, between-group comparisons were performed using independent-samples t-tests for normally distributed data and Mann–Whitney U tests for non-normally distributed data. Effect sizes were calculated for all primary between-group comparisons. Cohen’s d was used for parametric analyses, whereas effect size r was calculated for non-parametric analyses as r = Z/√N, where Z is the standardized test statistic and N is the total sample size. Ninety-five percent confidence intervals (95% CIs) were calculated for the effect sizes. Data in figures are presented as mean ± standard error (SE), and error bars indicate SE values.

Given the imbalance in sex distribution between the younger and older adult groups, additional exploratory analyses were conducted to examine the potential influence of sex on the primary outcome variables. Age group and sex were included as fixed factors in supplementary analyses. Because of the limited number of male participants in the older adult group, these analyses were interpreted cautiously.

To control for Type I error due to multiple comparisons, p-values for the nine primary muscle comparisons were adjusted using the Holm–Bonferroni sequential correction method [18]. Specifically, p-values were ranked from smallest to largest and sequentially compared with adjusted significance thresholds (α/(n − i + 1)). Analyses involving non-primary muscles were considered exploratory and were reported without correction.

As a sensitivity analysis, the same statistical procedures, including effect size estimation and multiple-comparison correction, were applied to RMS values calculated across the entire 5-second recording. The results were compared with those obtained from the primary analysis based on the selected 1-second epoch.

All analyses were performed using SPSS version 29.0.1.0 (IBM Corp., Armonk, NY, USA). A two-sided p-value < 0.05 was considered statistically significant.

### 2.5. Ethics

The present study was approved by the Research Ethics Committee of the Institute of Health and Sport Sciences, University of Tsukuba (Approval No. Tai-023–38). All participants were informed about the study purpose, the voluntary nature of participation, and their right to withdraw from the study at any time. All participants provided written informed consent. The study was conducted in accordance with the principles of the Declaration of Helsinki.

## 3. Results

### 3.1. Participant characteristics

Participant characteristics are shown in Table 2.

**Table 2:** Basic characteristics of the participants.

	Older Adults (n = 23)	Younger Adults (n = 20)	*p* value
Mean Age (years)	72.5 ± 6.8	21.0 ± 2.9	***
No. of Subjects (F/M)	19/4	9/11	*
Height (cm) (M ± SD)	156.5 ± 7.0	171.5 ± 9.2	*
Weight (kg) (M ± SD)	56.8 ± 8.4	59.2 ± 18.3	0.398
BMI (M ± SD)	23.2 ± 2.4	21.2 ± 3.7	0.054

***: p < .001, **: p < .01, *: < .05.

n.s: not significant.

Significant differences were observed in age (p < .001), sex distribution (p < .05), and height (p < .05), but not in weight (p = .398) or BMI (p = .200).

### 3.2. Task 1: Resting muscle activity

During the resting condition, older adults exhibited significantly higher muscle activity in several facial muscles, including the CS (p < .001, r = .553, 95% CI for r [.303,.732]), OOc (p = .001, r = .550, 95% CI for r [.299,.730]), LLSAN (p < .001, r = .550, 95% CI for r [.299,.730]), LLS (p = .001, r = .505, 95% CI for r [.241,.699]), ZM (p = .001, r = .486, 95% CI for r [.218,.687]), LAO (p = .002, r = .472, 95% CI for r [.199,.676]), and OOr (p = .008, r = .405, 95% CI for r [.119,.629]) compared with younger adults. No significant between-group differences were observed in FRO (p = .156) or DAO (p = .436) muscles (Fig 2).

**Fig 2:**
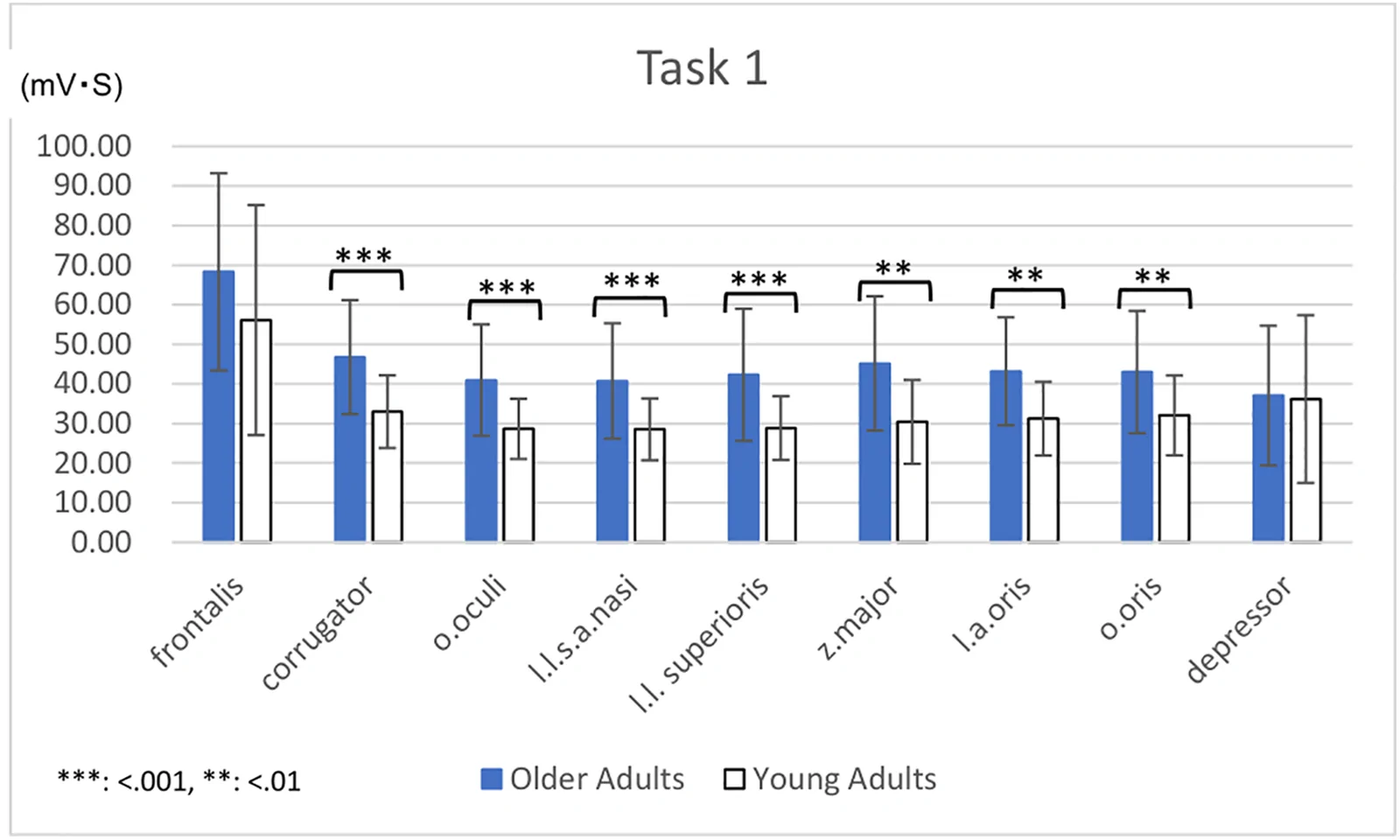
Neutral.

### 3.3. Tasks 2–10: Voluntary facial contractions

Normality testing of the primary muscle outcome for each task indicated that only the FRO was normally distributed; therefore, an independent-samples t-test was performed for this muscle. FRO activity was significantly higher in the younger adult group (M = 10.23, SD = 8.08) than in the older adult group (M = 4.60, SD = 2.60), t (22.43) = −2.986, p = .007, with a large effect size (Cohen’s d = 0.968, 95% CI for d [0.328, 1.597]).

Mann–Whitney U tests were conducted to compare facial muscle activity between the younger and older adult groups for the remaining eight target muscles. Muscle activity was significantly higher in the younger adult group than in the older adult group for six of the eight muscles: CS (U = 368, Z = 3.360, p < .001, r = .512, 95% CI for r [0.251, 0.704]), OOc (U = 426, Z = 4.772, p < .001, r = .728, 95% CI for r [0.547, 0.844]), LLSAN (U = 338, Z = 2.630, p = .009, r = .401, 95% CI for r [0.115, 0.626]), LLS (U = 405, Z = 4.261, p < .001, r = .650, 95% CI for r [0.434, 0.795]), ZM (U = 409, Z = 4.359, p < .001, r = .665, 95% CI for r [0.455, 0.804]), and OOr (U = 336, Z = 2.581, p = .010, r = .394, 95% CI for r [0.106, 0.621]). No significant between-group differences were observed for LAO (U = 295, Z = 1.583, p = .113, r = .241, 95% CI for r [−0.064, 0.505]) or DAO (U = 232, Z = 0.049, p = .961, r = .007, 95% CI for r [−0.294, 0.307]) (Figs 3–11).

**Fig 3:**
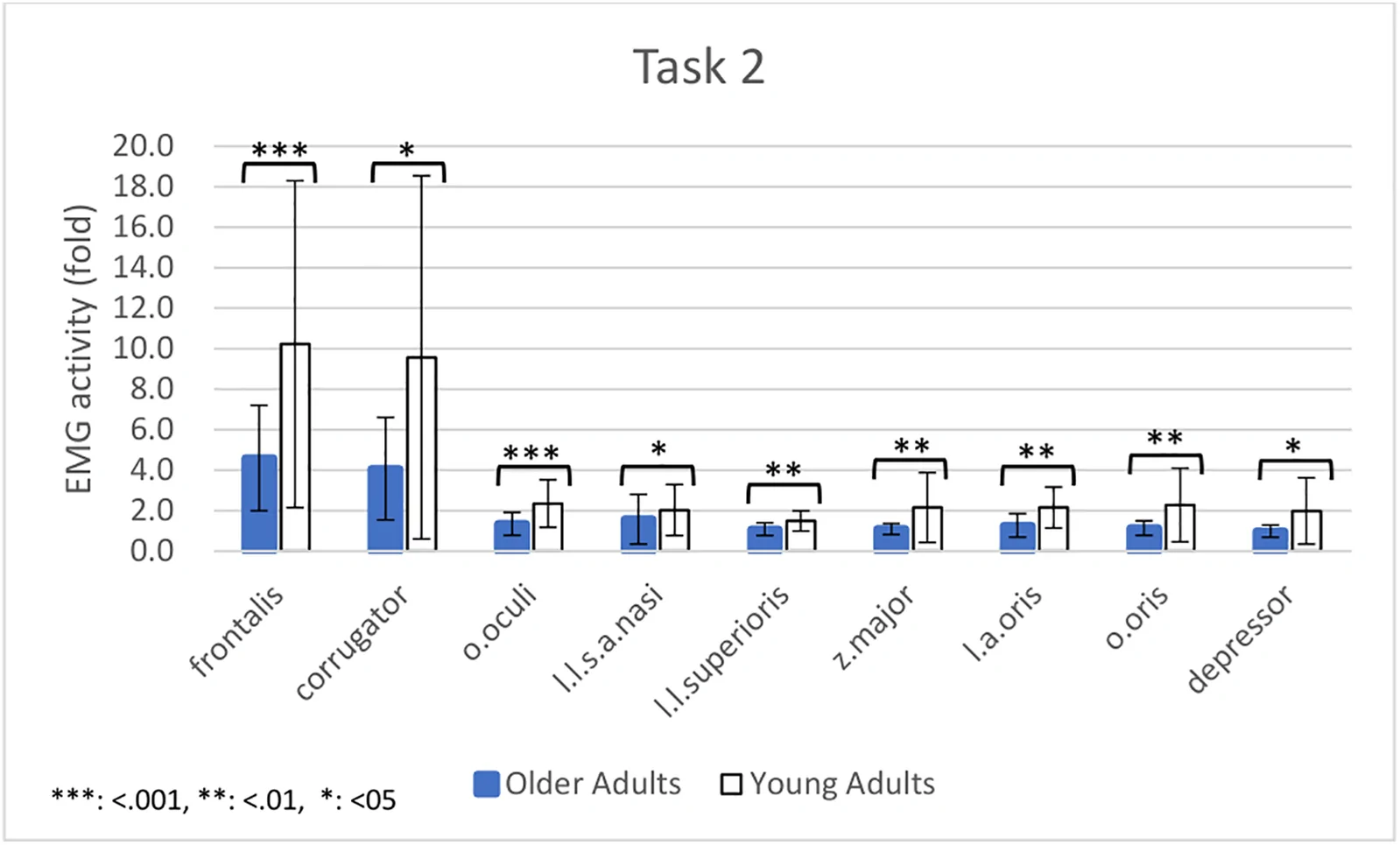
Frontalis muscle contraction.

**Fig 4:**
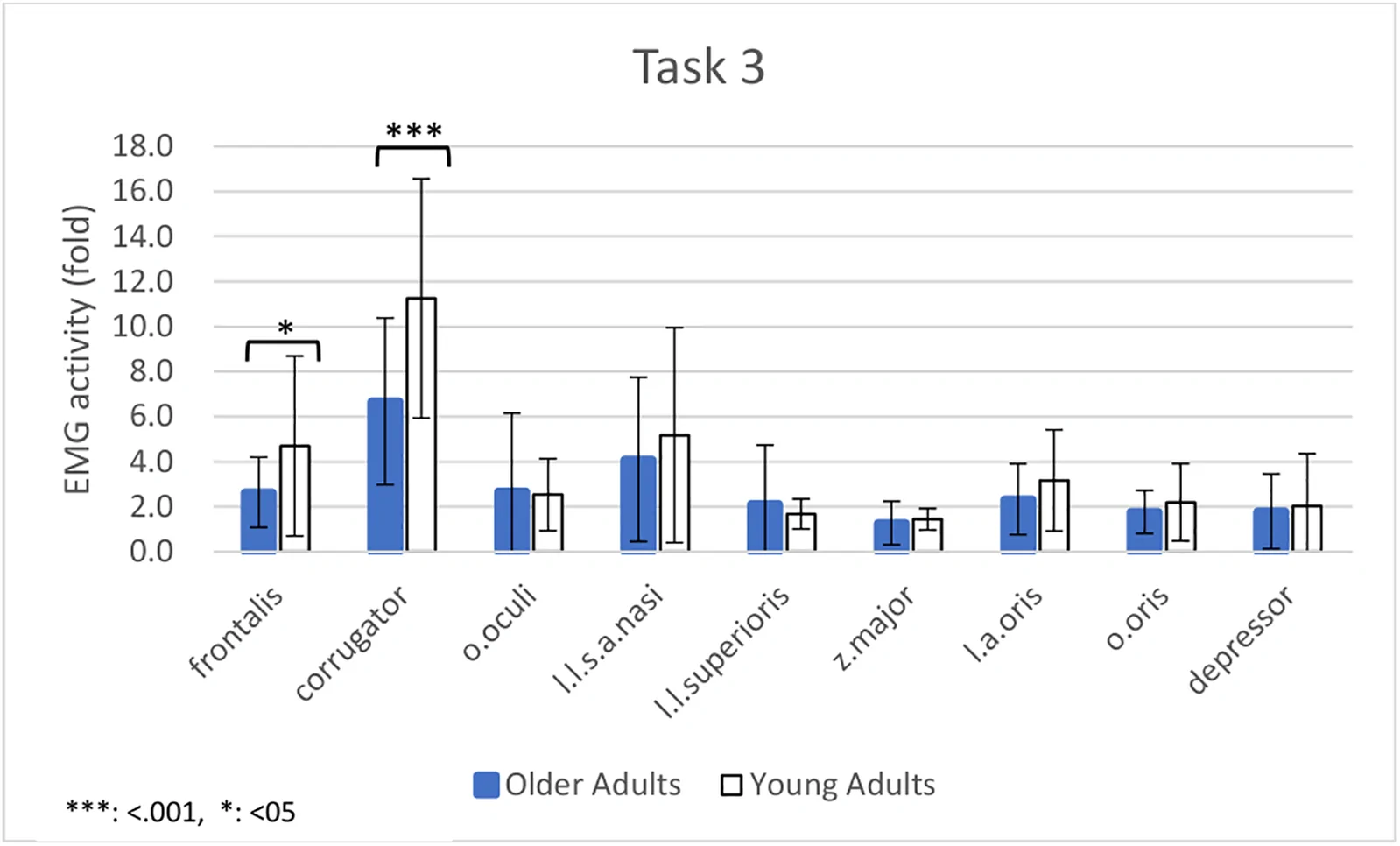
Corrugator supercilii muscle contraction.

**Fig 5:**
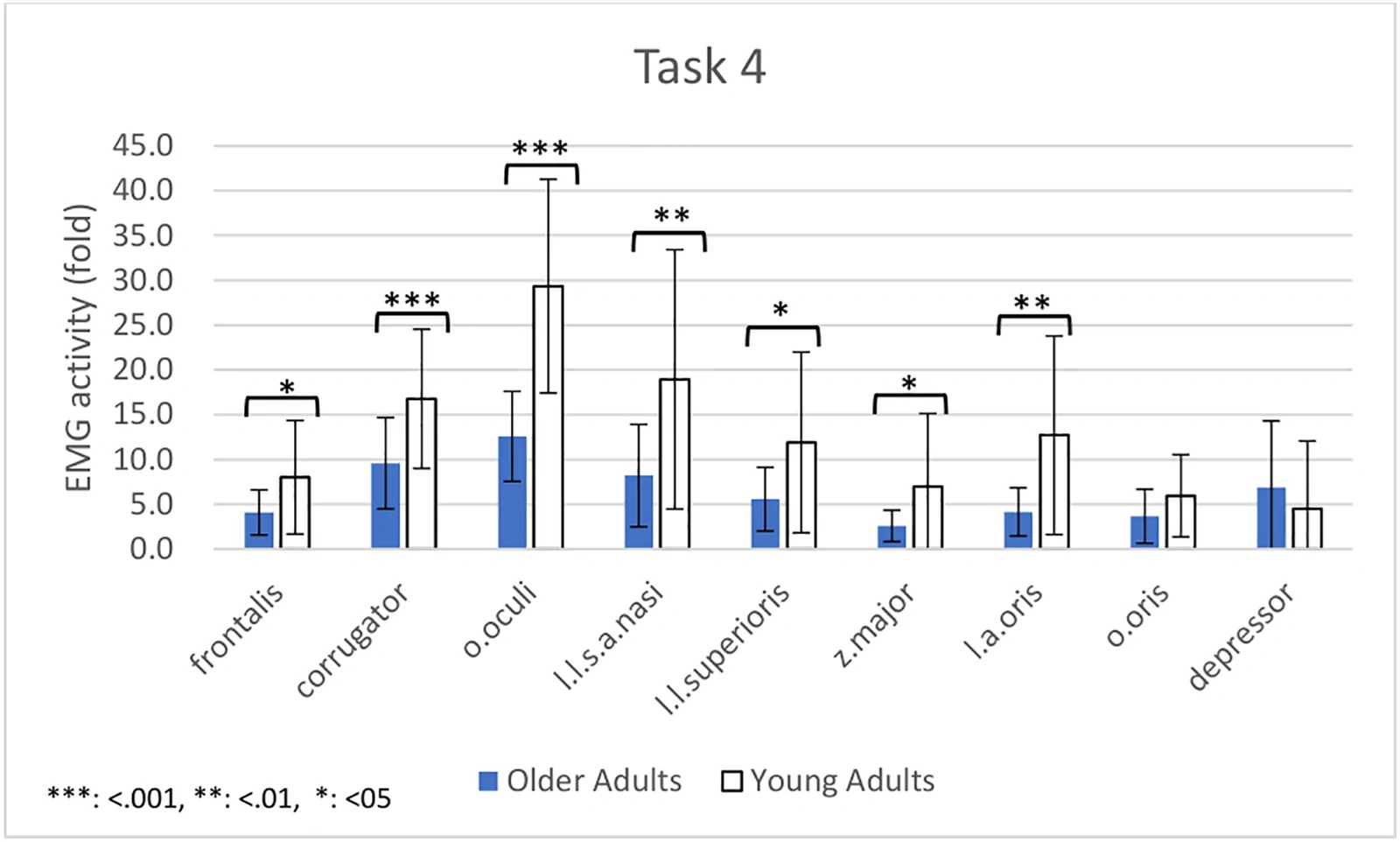
Orbicularis oculi muscle contraction.

**Fig 6:**
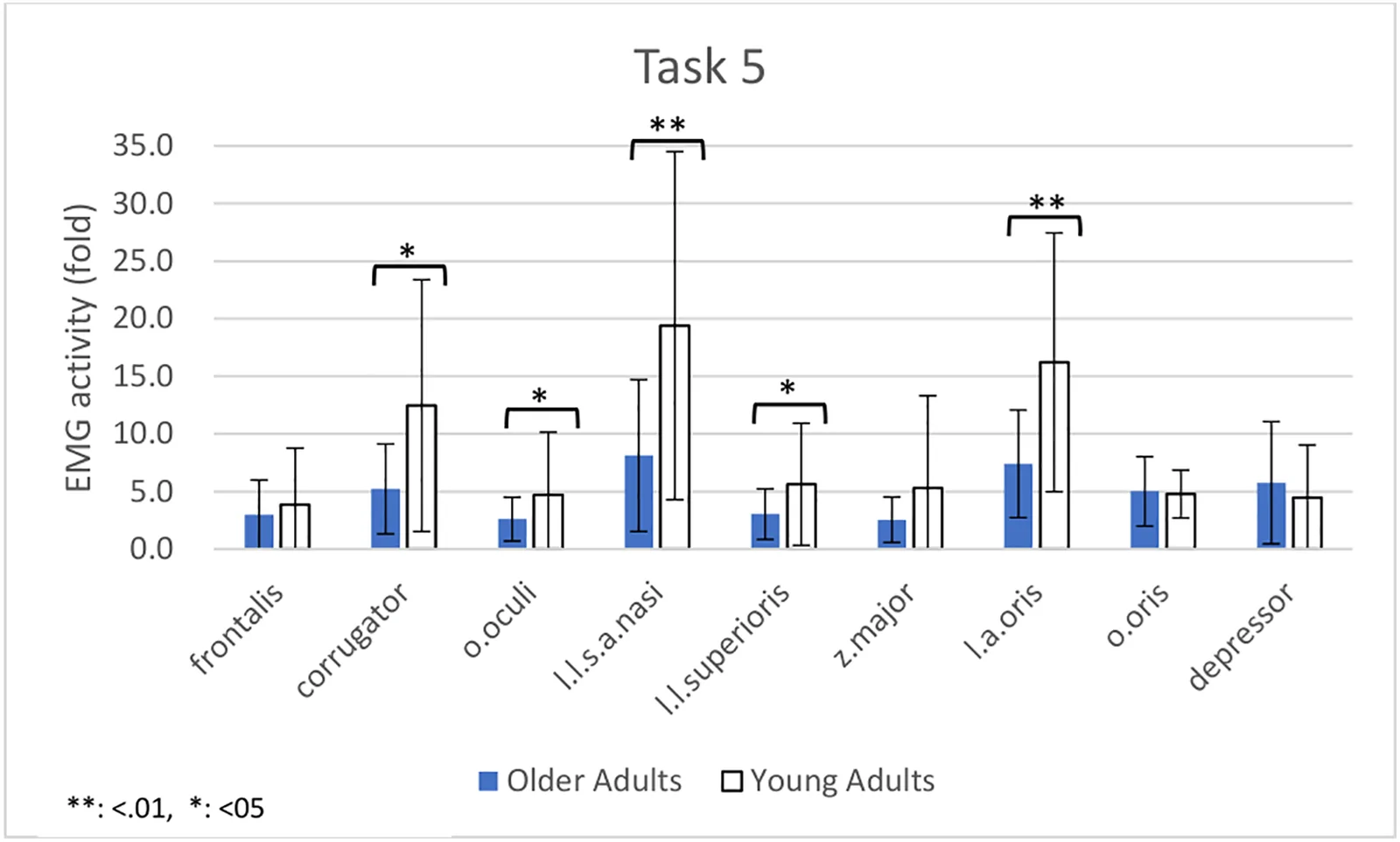
Levator labii superioris alaeque nasi muscle contraction.

**Fig 7:**
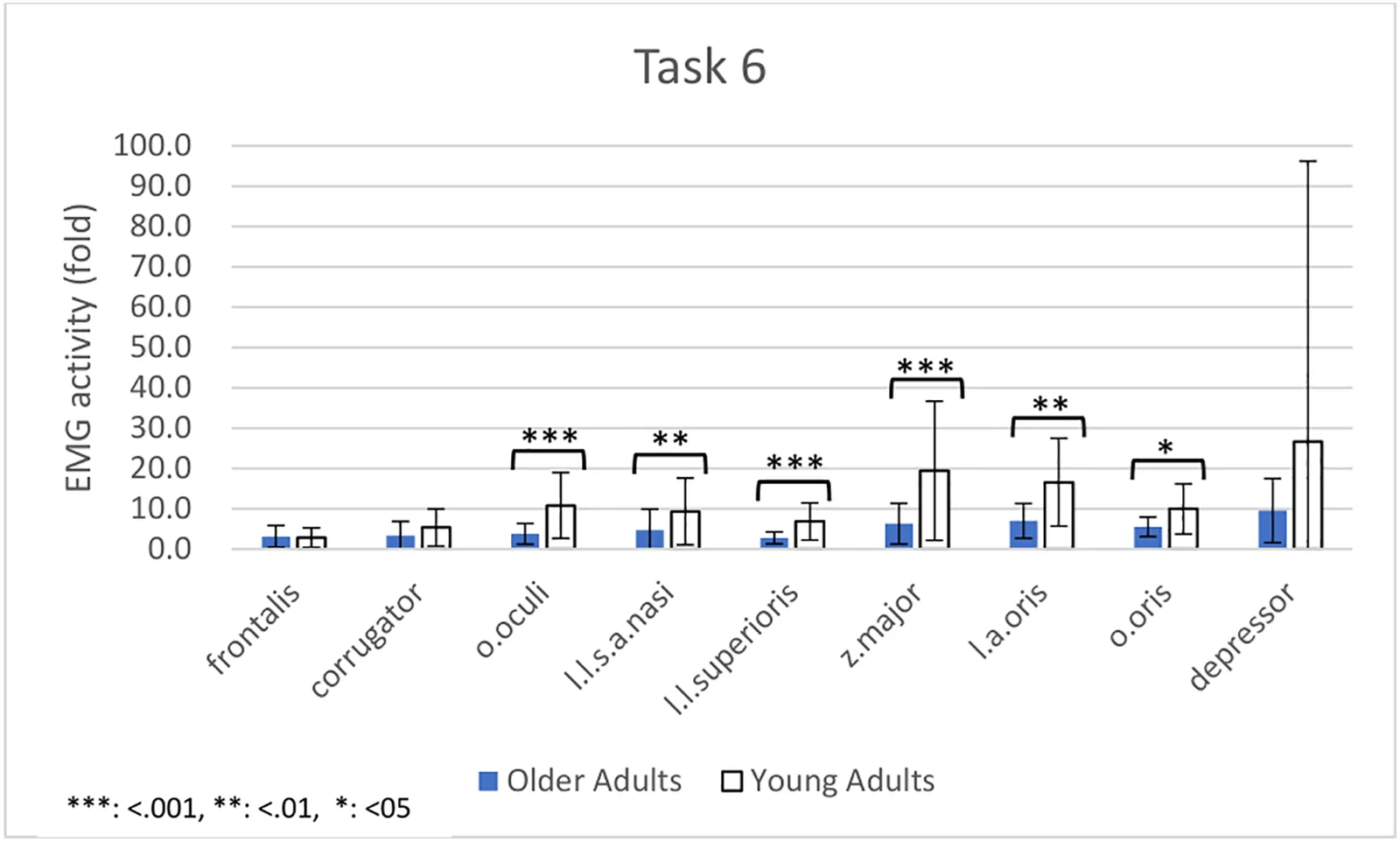
Levator labii superioris muscle contraction.

**Fig 8:**
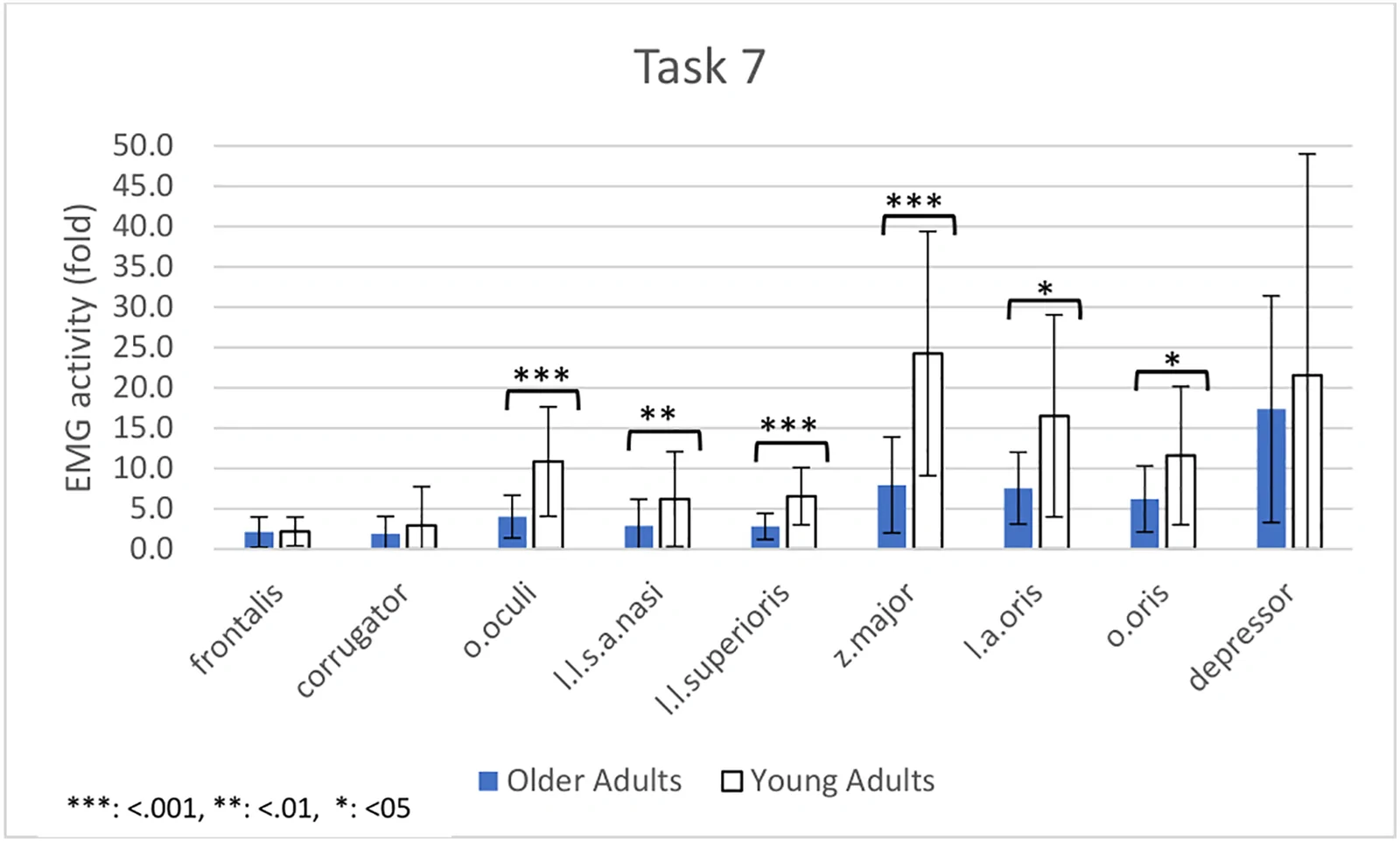
Zygomaticus major muscle contraction.

**Fig 9:**
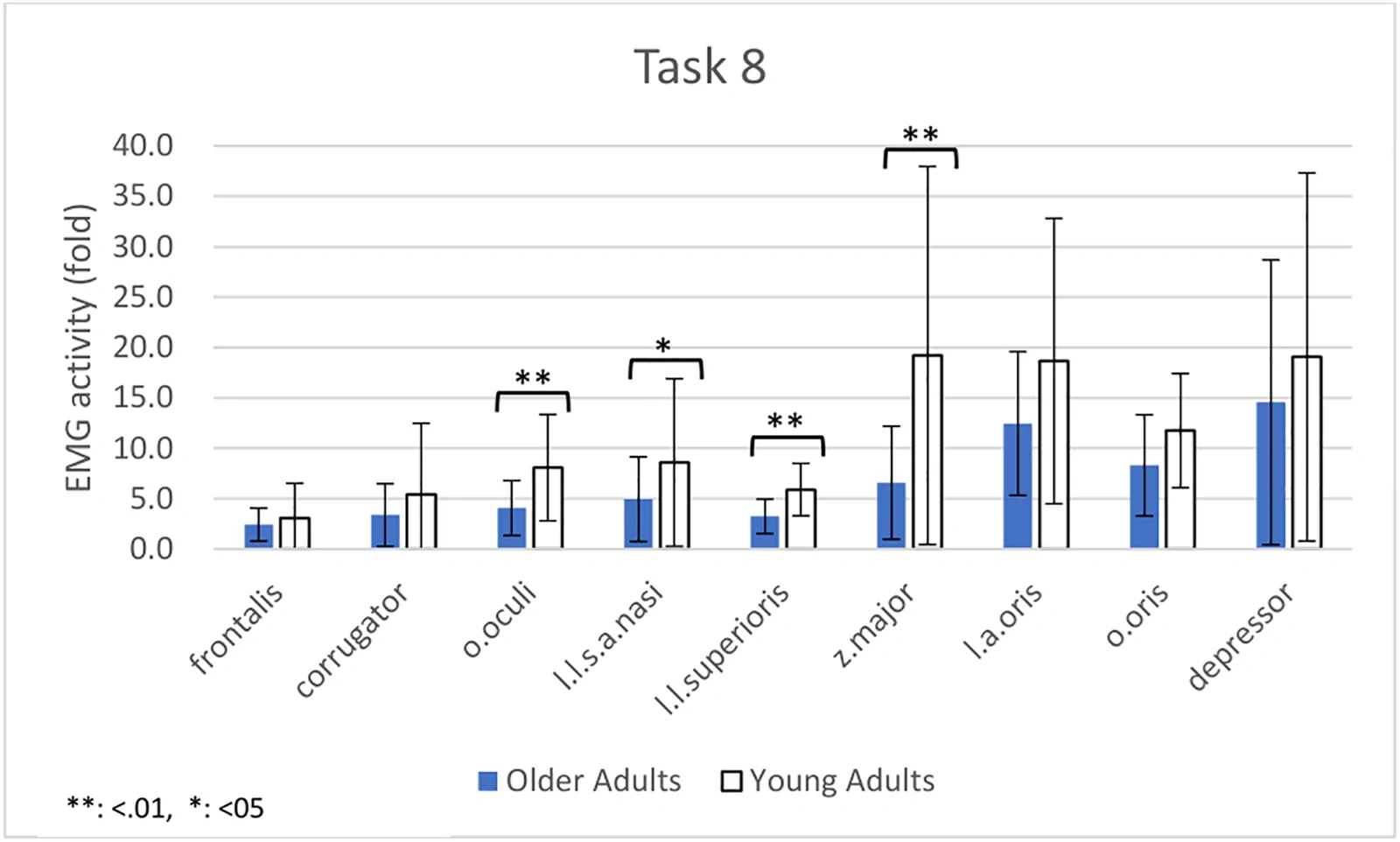
Levator anguli oris muscle contraction.

**Fig 10:**
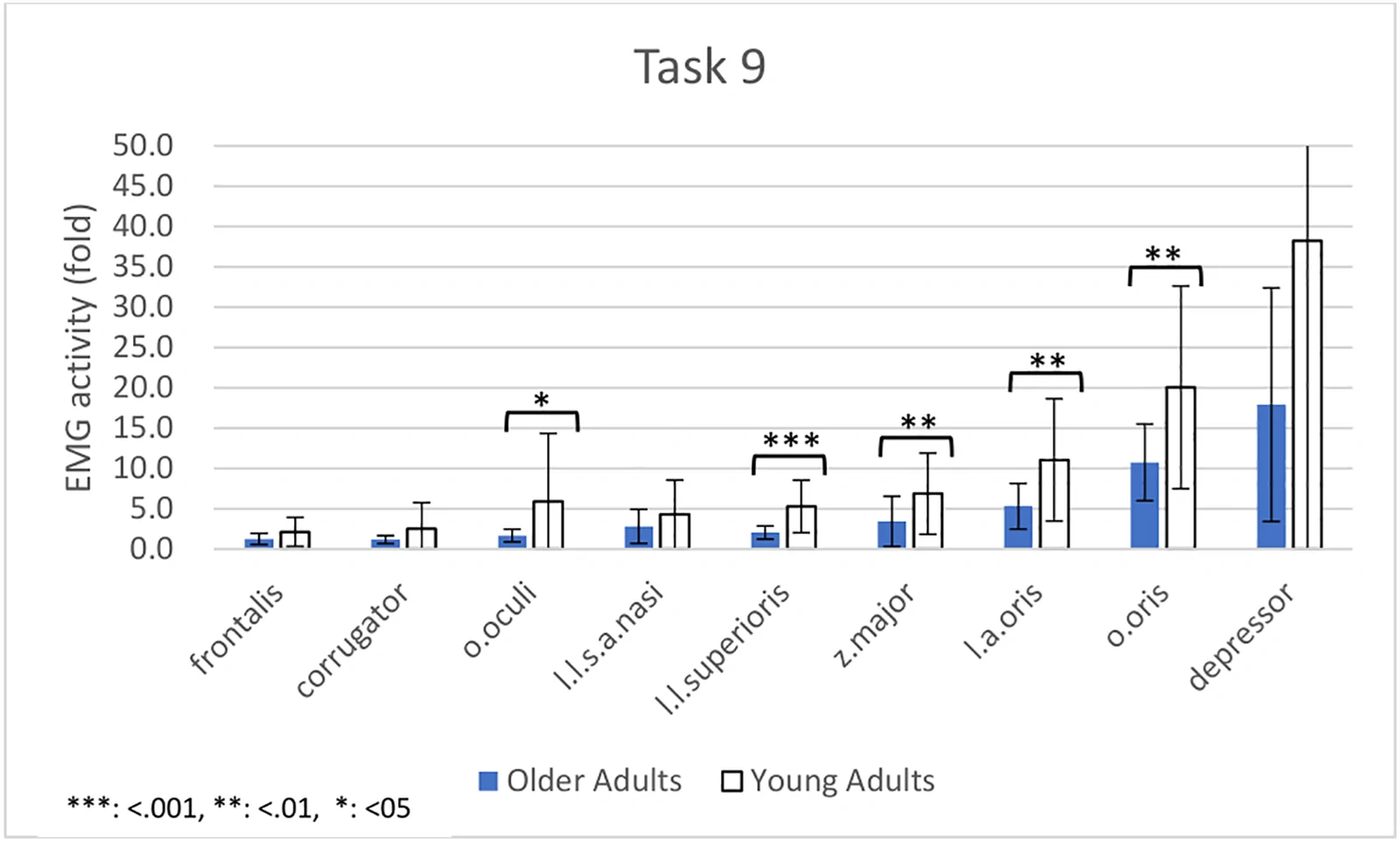
Orbicularis oris muscle contraction.

**Fig 11:**
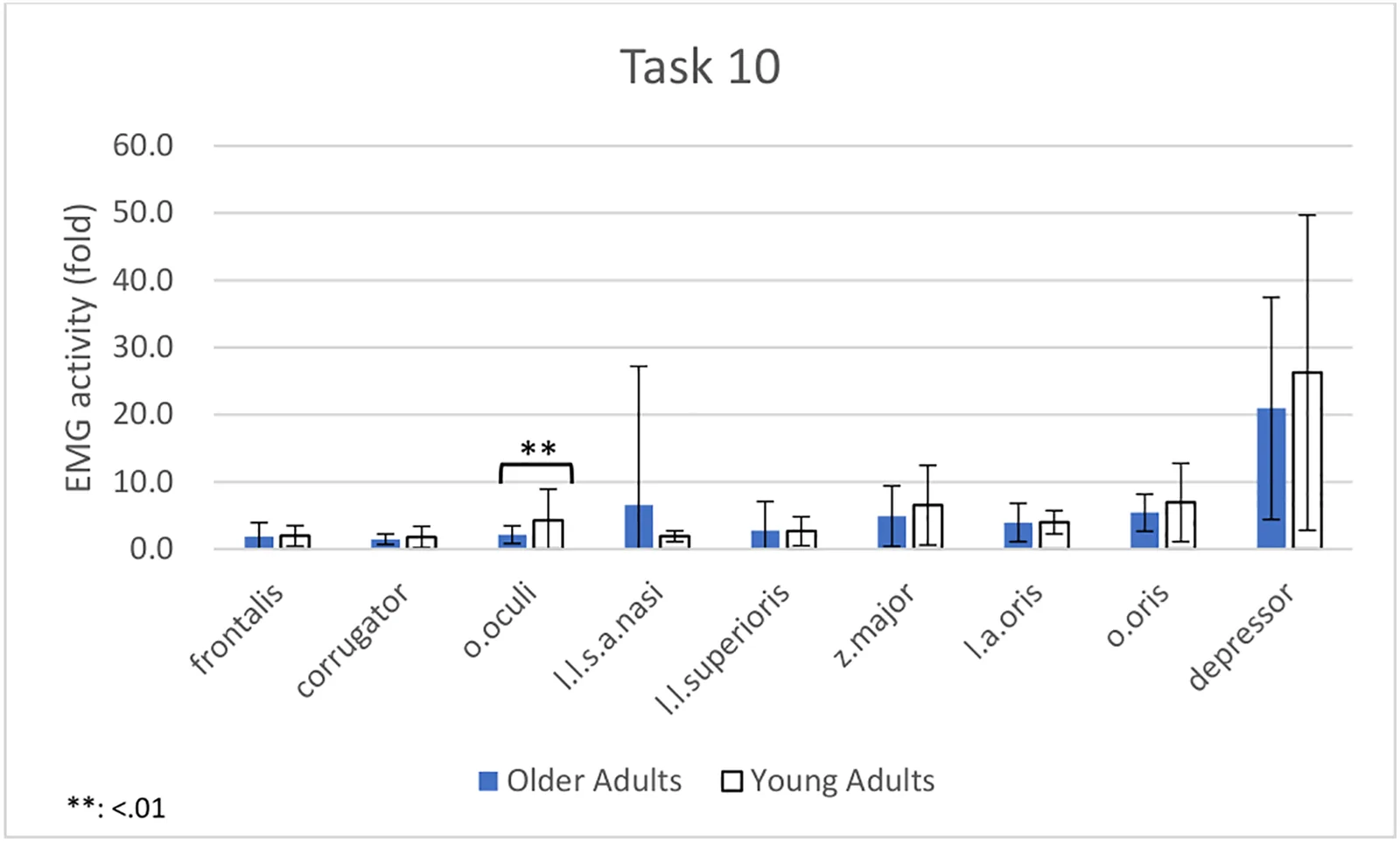
Depressor anguli oris muscle contraction.

Given the imbalance in sex distribution between the younger and older adult groups, additional exploratory analyses were conducted to examine the potential influence of sex on the primary outcome variables. Age group and sex were included as fixed factors in supplementary analyses. The primary between-group findings remained largely unchanged after accounting for sex. A significant sex effect was observed only for ZM activity (p = .030), whereas no significant sex effects were identified for the other primary muscle outcomes.

After applying the Holm correction across the nine primary muscle comparisons, significant group differences remained in the FRO (adjusted p = .008), CS (adjusted p = .005), OOc (adjusted p < .001), LLSAN (adjusted p = .027), LLS (adjusted p < .001), ZM (adjusted p < .001), and OOr (adjusted p < .001) muscles. In contrast, no significant differences were observed in the LAO (adjusted p = .226) or DAO (adjusted p = .961) muscles after correction.

### 3.4. Sensitivity analysis using the full 5-second recording

Sensitivity analyses were conducted using RMS amplitudes calculated across the entire 5-second recording for each task and muscle, rather than the selected 1-second epochs used in the primary analysis. The overall pattern of age-related differences was largely consistent with that observed in the primary analysis.

For the nine primary muscles, significant between-group differences were observed for eight muscles before Holm correction. Muscle activity was significantly higher in the younger adult group than in the older adult group for FRO (p < .001, r = .512, 95% CI for r [.251,.704]), CS (p < .001, r = .550, 95% CI for r [.299,.730]), OOc (p < .001, r = .757, 95% CI for r [.592,.862]), LLSAN (p < .001, r = .524, 95% CI for r [.265,.712]), LLS (p < .001, r = .653, 95% CI for r [.439,.797]), ZM (p < .001, r = .583, 95% CI for r [.343,.752]), and OOr (p = .003, r = .446, 95% CI for r [.168,.658]). LAO also showed a significant between-group difference (t(24.50) = −2.245, p = .034, Cohen’s d = −.722, 95% CI for d [−1.338, − .099]). No significant between-group difference was observed for DAO (p = .609, r = .078, 95% CI for r [−.228,.370]).

After applying the Holm correction across the nine primary muscle comparisons, significant group differences remained in the FRO (adjusted p = .009), CS (adjusted p = .009), OOc (adjusted p = .009), LLSAN (adjusted p = .009), LLS (adjusted p = .009), ZM (adjusted p = .009), and OOr (adjusted p = .009) muscles. In contrast, the difference in LAO was no longer significant after correction (adjusted p = .068), and no significant difference was observed for DAO (adjusted p = .609).

Thus, the primary findings were largely consistent when RMS amplitudes were calculated across the full 5-second recording rather than from a selected 1-second epoch.

## 4. Discussion

In Task 1, resting facial muscle activity was observed in both groups, and older adults exhibited significantly higher resting activity in several facial muscles than younger adults, suggesting that age-group differences may exist in baseline facial muscle activation. To our knowledge, few studies have examined differences in resting facial muscle activity between younger and older adults using sEMG. Interestingly, this finding differs from a previous facial sEMG study by Cotofana et al., which reported no significant differences between younger and older adults in baseline noise levels [14]. This discrepancy may be related to differences in study design, electrode placement, muscles assessed, or signal processing methods. One possible explanation for the higher resting activity observed in older adults is altered neuromuscular regulation. Experimental evidence from aged rats suggests that sympathetic modulation of skeletal muscle contractility is altered with aging, indicating that age-related changes in autonomic regulation may influence muscle contractile properties [19]. Although these findings were obtained from hindlimb muscles in an animal model and cannot be directly extrapolated to human facial muscles, altered autonomic or neuromuscular regulation may represent one possible mechanism underlying the differences in resting facial muscle activity observed in the present study. However, sEMG amplitude does not directly reflect muscle force or muscle mass and may be influenced by factors such as subcutaneous tissue characteristics, electrode placement, and motor unit recruitment patterns. Therefore, the present findings should be interpreted with caution. These methodological and physiological factors may be particularly relevant to facial muscles because they are anatomically small and require highly coordinated activation. In addition, Le Louarn et al. proposed that repeated contractions of facial mimetic muscles may contribute to structural facial aging by altering resting muscle morphology and tone [20]. Although their hypothesis was based on anatomical and radiological observations rather than electrophysiological measurements, these structural changes may provide one possible explanation for the elevated resting activity observed in older adults in the present study. However, the underlying mechanisms remain unclear. Increased resting activity may also be related to age-associated changes in muscle tone regulation. Nevertheless, interpretations involving sympathetic-mediated mechanisms should be made cautiously because no direct physiological measurements were obtained in this study.

In Tasks 2–10, muscle activity during voluntary facial contractions was generally lower in older adults than in younger adults in the muscles corresponding to the intended facial movements. These findings are broadly consistent with those of Cotofana et al., who reported an overall tendency toward reduced absolute facial muscle signal with older age, with a statistically significant reduction observed in ZM in older adults [14]. The present findings extend these previous observations by demonstrating significant differences between younger and older adults across a broader range of facial muscles and specific voluntary facial movement tasks. In addition, differences were observed in adjacent muscle regions, suggesting that age-group differences may involve not only the magnitude of facial muscle activation but also coordinated activation patterns among neighboring muscles.

In Task 5, increased activity in adjacent muscle regions may indicate differences in coordination patterns during facial movements. Anatomically, the LLSAN is connected to the LAO [21], and coordinated activation between these muscles may contribute to the observed findings.

In Task 6, although a significant group difference was observed in LLS, relatively greater activity was also observed in adjacent muscles. This may indicate that the instructed movement (“raise the cheeks straight upward”) was difficult to isolate and may have elicited broader facial muscle recruitment patterns. A similar pattern was observed for the movement targeting the LAO in Task 8, which showed a tendency toward lower activity in older adults, although the between-group difference did not reach statistical significance in the primary analysis. Notably, a significant group difference was observed in the sensitivity analysis using 5-second RMS amplitudes, although this did not survive Holm correction. The fact that a significant group difference emerged only when RMS amplitudes were averaged across the full 5-second recording period, but not within a stable 1-second epoch, may reflect greater within-trial fluctuation in LAO activation in older adults compared with younger adults. These observations raise the possibility that facial muscles less frequently recruited during habitual expressive movements may exhibit different age-group patterns in motor activation. Facial expressions such as smiling or frowning are deeply integrated into spontaneous daily behavior, whereas some facial muscles may be less frequently recruited independently during everyday facial movements. Reduced habitual recruitment could potentially contribute to differences in neuromuscular capacity through a disuse-related mechanism. However, this interpretation remains speculative because habitual facial muscle use and longitudinal changes in muscle function were not assessed in the present cross-sectional study.

Notably, age-group differences remained significant for most primary muscle comparisons after Holm correction, suggesting that the observed findings were robust to adjustment for multiple comparisons. Importantly, the overall pattern of age-group differences was also maintained in the sensitivity analysis using RMS amplitudes calculated across the full 5-second recording, supporting the robustness of the findings to the method used to define the analysis epoch. In contrast, an additional exploratory analysis indicated a significant sex effect only for ZM activity, whereas the other primary outcomes remained largely unchanged after accounting for sex.

These findings suggest that differences in facial muscle function between younger and older adults may extend beyond muscle activity during specific voluntary contractions and may also involve resting muscle activation and coordinated recruitment during facial movements. Because facial muscles contribute not only to emotional expression but also to communication, oral-related movements, and social interaction, age-associated differences in facial motor function may have broader implications for healthy aging. Furthermore, assessing facial muscle function itself—beyond observable facial expressions—may offer a potentially useful avenue for investigating age-associated neuromuscular decline. Given that frailty is characterized by a progressive loss of physiological reserve and functional capacity, facial muscle motor capacity may warrant investigation as a potential marker of broader neuromuscular aging and functional decline. However, longitudinal and functional studies combining electrophysiological, structural, and behavioral assessments are needed to determine whether facial muscle function can serve as a clinically meaningful indicator of age-related neuromuscular decline and to clarify its potential relevance to frailty.

## 5. Limitations

Several limitations of this study should be acknowledged. First, although task performance was standardized through verbal instructions, demonstrations, practice trials, and video confirmation, we could not objectively quantify facial contraction intensity. Therefore, inter-individual variability in task effort may have influenced the observed EMG amplitudes.

Second, because older adults exhibited higher resting activity in several muscles, normalization using resting values may have influenced the relative magnitude of the between-group differences observed during voluntary tasks. Although raw RMS values were provided in the Supplementary Material for transparency, future studies should evaluate alternative normalization approaches for facial sEMG.

Third, there was an imbalance in sex distribution between the younger and older adult groups. The older group consisted predominantly of female participants, which may have contributed to the observed differences in facial muscle activity. Although exploratory analyses including sex indicated that the primary age-group findings remained largely unchanged, residual confounding by sex cannot be completely excluded. In addition, educational background and other psychosocial characteristics that may influence facial behavior were not systematically assessed. Future studies should incorporate more comprehensive participant profiling and balanced samples to better account for potential confounding factors.

Fourth, we used relatively small electrodes (outer diameter: 5 mm), which are smaller than those commonly used in conventional sEMG assessments of larger skeletal muscles, to minimize potential crosstalk in facial sEMG recordings. However, because facial muscles are anatomically small and closely adjacent, complete elimination of crosstalk cannot be guaranteed. In addition, the presence of electrodes on the face may have altered participants’ natural facial movements. Although the skin was cleaned with alcohol prior to electrode placement to reduce impedance, skin impedance was not directly measured. Because a single recording trial was obtained for each facial movement task, trial-to-trial variability could not be evaluated. Although stable 1-second epochs were visually confirmed and selected for the primary analysis, this approach may introduce some degree of subjectivity. To address the potential influence of epoch selection, we additionally performed sensitivity analyses using RMS amplitudes calculated across the entire 5-second recording, and the overall pattern of findings remained largely consistent. Nevertheless, future studies should incorporate repeated trials and more systematic artifact-rejection procedures to improve measurement reliability and reduce potential selection bias. The relatively small sample size may also have limited statistical power and increased uncertainty in the estimates; therefore, studies with larger samples are warranted.

Fifth, this study was conducted exclusively in Japanese participants; therefore, comparative studies involving participants from other populations are needed to determine the generalizability of the findings.

Finally, this study included only younger and older adults. The absence of middle-aged participants limited our ability to characterize changes in facial muscle activity across the adult lifespan. Future studies should include middle-aged adults to better understand age-associated changes across the lifespan. In addition, longitudinal studies are warranted to directly examine the trajectory of facial muscle activity across the lifespan, to clarify whether the observed between-group differences reflect progressive age-related changes, and to better understand the underlying mechanisms.

Beyond the limitations specific to sEMG methodology, the broader challenge of objectively assessing facial muscle function in clinical and community settings remains. Facial sEMG, while informative, requires specialized equipment and technical expertise, which may limit its widespread application. The development of simpler and more accessible methods for evaluating facial muscle function — that go beyond the observation of facial expressions — would be an important step toward enabling routine assessment of age-related neuromuscular changes and informing preventive strategies for functional decline.

## 6. Conclusions

In this cross-sectional study, measurements of facial muscle activity in younger and older Japanese adults demonstrated that:

(1) during the neutral facial condition, facial muscle activity was significantly higher in older adults than in younger adults; and
(2) during voluntary facial movements intended to engage specific facial muscles as functional units, facial muscle activity in multiple muscles was significantly lower in older adults than in younger adults.

These findings suggest that facial muscle activation patterns — reflecting not merely movement execution but the functional capacity of facial muscles — may change with aging. However, because of the cross-sectional nature of the study, causal relationships between aging and facial muscle activity cannot be established. Longitudinal studies are warranted to clarify the trajectory of age-related changes in facial muscle function.

## Supporting information

S1 File S1 Table. Raw RMS values for the FRO movement task across facial muscles. S2 Table. Raw RMS values for the CS movement task across facial muscles. S3 Table. Raw RMS values for the OOc movement task across facial muscles. S4 Table. Raw RMS values for the LLSAN movement task across facial muscles. S5 Table. Raw RMS values for the LLS movement task across facial muscles. S6 Table. Raw RMS values for the ZM movement task across facial muscles. S7 Table. Raw RMS values for the LAO movement task across facial muscles. S8 Table. Raw RMS values for the OOr movement task across facial muscles. S9 Table. Raw RMS values for the DAO movement task across facial muscles.
(ZIP)
